# Supplementary material for: A Reconfigurable Polarimetric Photodetector Based on the MoS2/PdSe2 Heterostructure with a Charge-Trap Gate Stack
Source: Nanomaterials (Basel). 2024 Dec 1;14(23):1936. doi: 10.3390/nano14231936 (PMC11643881; doi:10.3390/nano14231936)
Supplement: Supplementary file 1 [file nanomaterials-14-01936-s001.zip › nanomaterials-3316386-supplementary.pdf]

Supplementary Materials

A Reconfigurable Polarimetric Photodetector Based on the MoS_2/$PdSe_2$ Heterostructure with a Charge-Trap Gate Stack

Xin Huang [1,2,*,†], Qinghu Bai [1,2,†], Yang Guo [1,2], Qijie Liang [3], Tengzhang Liu [4,5], Wugang Liao [4,5], Aizi Jin [1], Baogang Quan [1], Haifang Yang [1], Baoli Liu [1,3,6] and Changzhi Gu [1,2,*]

[1] Beijing National Laboratory for Condensed Matter Physics, Institute of Physics, Chinese Academy of Sciences, Beijing 100190, China; baiqinghu@iphy.ac.cn (Q.B.); yangguo@aphy.iphy.ac.cn (Y.G.); azjin@iphy.ac.cn (A.J.); quanbaogang@iphy.ac.cn (B.Q.); hfyang@iphy.ac.cn (H.Y.); blliu@iphy.ac.cn (B.L.)
[2] School of Physical Sciences, CAS Key Laboratory of Vacuum Physics, University of Chinese Academy of Sciences, Beijing 100190, China
[3] Songshan Lake Materials Laboratory, Dongguan 523808, China; liangqijie@sslab.org.cn
[4] State Key Laboratory of Radio Frequency Heterogeneous Integration, Shenzhen University, Shenzhen 518060, China; 2200434018@email.szu.edu.cn (T.L.); wgliao@szu.edu.cn (W.L.)
[5] College of Electronics and Information Engineering, Shenzhen University, Shenzhen 518060, China
[6] CAS Center for Excellence in Topological Quantum Computation, CAS Key Laboratory of Vacuum Physics, University of Chinese Academy of Sciences, Beijing 100190, China
* Correspondence: xinhuang@iphy.ac.cn (X.H.); czgu@iphy.ac.cn (C.G.)
† These authors contributed equally to this work.

Supplementary Section S1

For device fabrication, a silicon wafer with a 300-nm-thick thermally grown SiO_2 was chosen as the starting substrate. Charge-trap stack of Al_2O_3/HfO_2/ Al_2O_3 (AHA) (6 nm/8 nm/32 nm) were deposited via atomic layer deposition (ALD). During the ALD process, trimethylaluminum and trtrakis (ethyl-methylamido) hafnium were reacted with water under 200℃ for Al_2O_3 and HfO_2, respectively. Then the drain-source electrodes of the device were fabricated by e-beam lithography (EBL) and Ti/Au (5 nm/60 nm) were deposited by e-beam evaporation.

Thin flakes of MoS_2 and $PdSe_2$ were mechanically exfoliated from bulk crystals (HQ Graphene) and prepared with PDMS stamp by dry-transfer technique. The MoS_2 flake with proper size was firstly exfoliated by blue tape (Nitto Processing Tape) and transferred to the PDMS stamp, which was then attached to a glass slide and aligned to the pattered Au electrode. For the first transfer process, a part of the chosen MoS_2 flake is coated on the Au electrode, while a large part of the MoS_2 flake is transferred onto the AHA substrate. For the second transfer process, the $PdSe_2$ flake was aligned on top of the MoS_2 flake by the same procedure. Part of the $PdSe_2$ flake was also contacted with another Au electrode for following electrical characterization.

The thickness of the nanosheets was determined with atomic force microscopy (Bruker Dimension Icon). As shown in Figure S1, the thickness of MoS_2 and $PdSe_2$ was 5 nm and 15 nm, respectively.

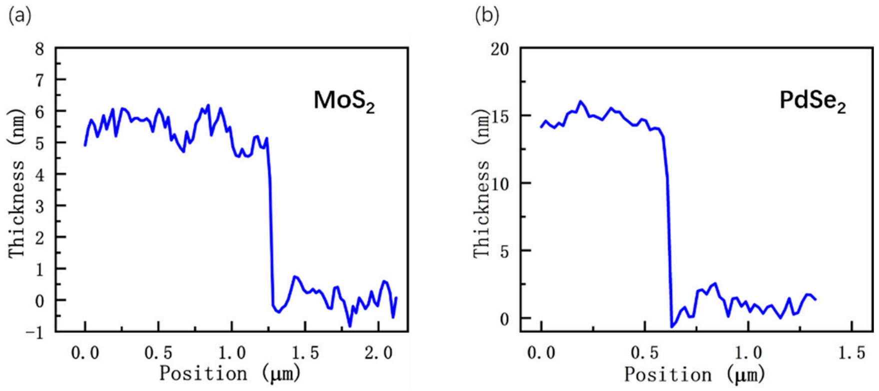

Figure S1. Thickness of (a) MoS_2 and (b) $PdSe_2$ measured by atomic force microscopy.

Supplementary Section S2

Raman spectra were collected by a confocal Raman microscopy system (Horiba LabRam HR Evolution) with excitation 532 nm laser. The electrical and photoelectric characteristics of the devices were measured in ambient conditions using a probe station and a semiconductor analyzer (4200SCS, Keithley). The photoelectric measurements were conducted on a self-built optical platform. The angle-resolved photocurrent was measured using a half-wave plate and a polarizer, with the laser polarization direction determined by the polarizer and modulated to achieve linearly polarized light. The polarization angle of the incident light was adjusted by rotating the half-wave plate without affecting the laser power.

Supplementary Section S3

Raman spectroscopy provides rich information of phonon vibration and crystal orientation identification. As mentioned above, $PdSe_2$ holds a puckered pentagonal structure, and its phonon vibrational anisotropy can be diagnosed by angle-resolved polarized Raman spectroscopy (ARPRS). In order to reveal the crystalline orientation of $PdSe_2$ by ARPRS, the angle-resolved Raman spectrum of the typical 143 cm^{-1} peak was measured under parallel polarization configuration and it is fitted in the polar plot in Figure S2. The Raman intensity at a definite angle θ can be fitted as: $\delta_\theta = \delta_b cos^2(\theta+\varphi) + \delta_a sin^2(\theta+\varphi)$, where δ_b or δ_a is the Raman intensity along b-axis or a-axis, and φ is fitting parameters.

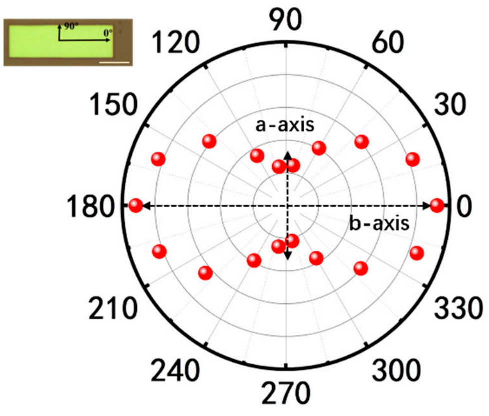

Figure S2. Angle-resolved polarized Raman spectroscopy of $PdSe_2$ flake.

Supplementary Section S4

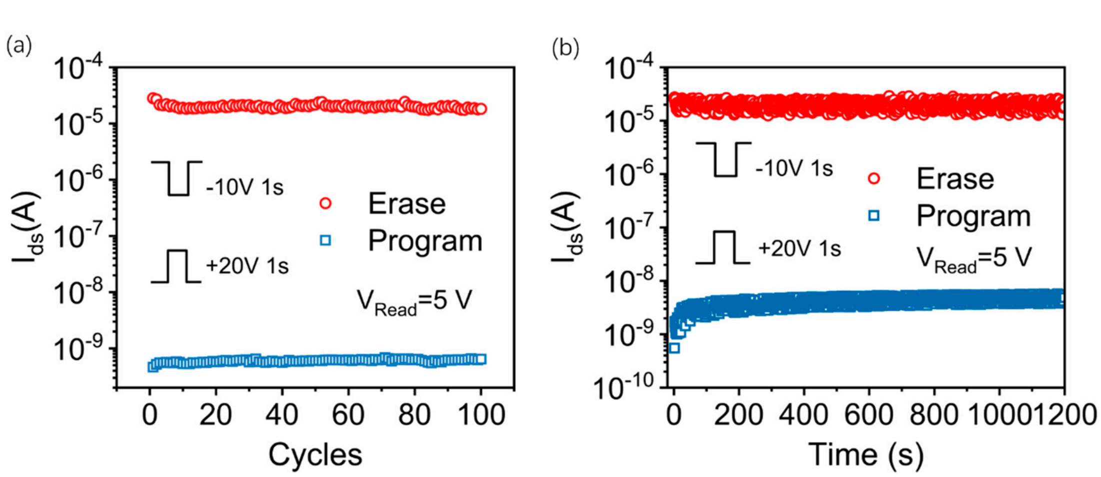

Figure S3. Endurance of the $MoS_2/PdSe_2$ memory. (a) Endurance of the memory device for 100 cycles with the program/erase voltage being +20 V, 1 s and −10 V, 1 s. (b) The stability of the program/erase state of the device after programming at +20 V, 1 s duration and erasing at −10 V, 1 s duration (V_{ds} = -1 V).

Supplementary Section S5

Considering the degeneration of 2D flakes and environment noise to photocurrent measurement, we continue to optimize the device and measurement setup for better performance. 10 nm-thickness protection layer of Al_2O_3 was deposited on top of $MoS_2/PdSe_2$ heterostructure to avoid the degeneration of device during the measurement. Furthermore, under the linear-polarized laser chopped by an optical chopper at 1 kHz frequency, the photocurrents of $MoS_2/PdSe_2$ photodetector with erase and program state were measured by a lock-in amplifier to reduce noise, as shown in Figure S4.

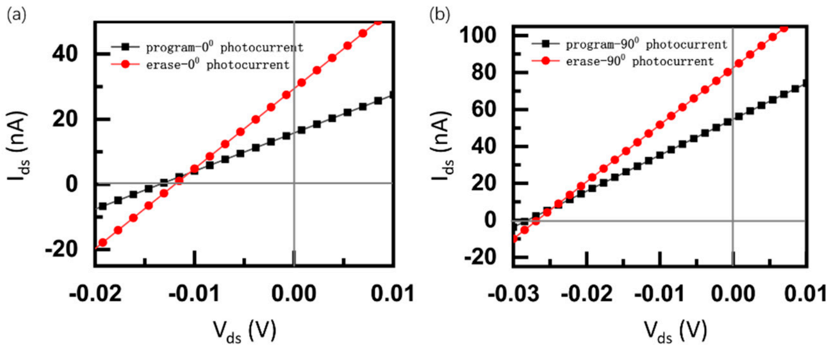

Figure S4. The polarization-modulated photovoltaic behavior of $MoS_2/PdSe_2$ photodetector: (a) and (b) Short-circuit current I_{sc} and open-circuit voltage V_{oc} of $MoS_2/PdSe_2$ photodetector under program and erase state, respectively.

Supplementary Section S6

To evaluate the detection limit of our device, we then analyze noise equivalent power (NEP) and detectivity of this photodetector. The NEP and detectivity are estimated and shown in Figure S5 according to the following equations:

$$NEP = \frac{noise}{Responsivity}$$

$$Detectivity = \frac{\sqrt{A}}{NEP}$$

where $noise$ is determined by the dark current according to the equation: $noise = \sqrt{2qI_{dark}\Delta f}$, where I_{dark} and Δf is dark current and bandwidth. A is the device active area.

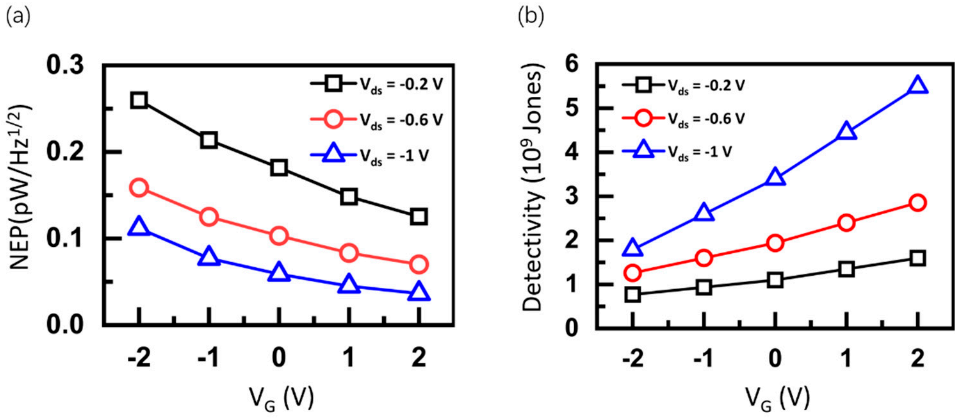

Figure S5. The NEP and detection limit of $MoS_2/PdSe_2$ photodetector. (a) NEP and (b) detectivity tuned by the gate voltage, indicating the detection limit of our photodetector.
